# A novel magnetic compression technique for establishment of a vesicovaginal fistula model in Beagle dogs

**DOI:** 10.1038/s41598-024-55466-y

**Published:** 2024-04-04

**Authors:** Miaomiao Zhang, Yingying Zhuang, Jianqi Mao, Linxin Shen, Xin Lyu, Yi Lyu, Xiaopeng Yan

**Affiliations:** 1 Department of Hepatobiliary Surgery, The First Affiliated Hospital of Xi’an Jiaotong University, No. 277 West Yanta Road, Xi’an, 710061 Shaanxi China; 2 Shaanxi Provincial Key Laboratory of Magnetic Medicine, The First Affiliated Hospital of Xi’an Jiaotong University, Xi’an, 710061 Shaanxi China; 3 Obstetrics Department, Baoji Central Hospital, Baoji, Shaanxi China; 4 Zonglian College, Xi’an Jiaotong University, Xi’an, Shaanxi China; 5 Department of Pulmonary and Critical Care Medicine, The Second Affiliated Hospital of Xi’an Jiaotong University, No. 3 Shang Qin Road, Xincheng District, Xi’an, 710004 Shaanxi China

**Keywords:** Magnetic compression technique, Vesicovaginal fistula, Magnetosurgery, Beagle dog, Animal model, Urinary tract, Bladder disease, Bladder, Experimental models of disease

## Abstract

Vesicovaginal fistula lacks a standard, established animal model, making surgical innovations for this condition challenging. Herein, we aimed to non-surgically establish vesicovaginal fistula using the magnetic compression technique, and the feasibility of this method was explored using eight female Beagle dogs as model animals. In these dogs, cylindrical daughter and parent magnets were implanted into the bladder and vagina, respectively, after anesthesia, and the positions of these magnets were adjusted under X-ray supervision to make them attract each other, thus forming the structure of daughter magnet-bladder wall-vaginal wall-parent magnet. Operation time and collateral damage were recorded. The experimental animals were euthanized 2 weeks postoperatively, and the vesicovaginal fistula gross specimens were obtained. The size of the fistula was measured. Vesicovaginal fistula was observed by naked eye and under a light microscope. Magnet placement was successful in all dogs, and remained in the established position for the reminder of the experiment. The average operation time was 14.38 min ± 1.66 min (range, 12–17 min). The dogs were generally in good condition postoperatively and were voiding normally, with no complications like bleeding and urine retention. The magnets were removed from the vagina after euthanasia. The vesicovaginal fistula was successfully established according to gross observation, and the fistula diameters were 4.50–6.24 mm. Histological observation revealed that the bladder mucosa and vaginal mucosa were in close contact on the internal surface of the fistula. Taken together, magnetic compression technique is a simple and feasible method to establish an animal model of vesicovaginal fistula using Beagle dogs. This model can help clinicians study new surgical techniques and practice innovative approaches for treating vesicovaginal fistula.

## Introduction

Vesicovaginal fistula (VVF) is a pathologic passage between the bladder and vagina, and its pathogenesis is mainly related to iatrogenic injury, including gynecological surgery and pelvic tumor radiation treatment injury^1–3^. VVF results in continuous wetness of the genital area, undesirable odor, and vaginal and bladder infections, resulting in severe displeasure and compromised quality of life^3^. Most patients with VVF need surgical treatment, and although there are various surgical methods^4–6^, each has its own shortcomings and indications. There is no standardized and unified surgical method for VVF. A large animal model of VVF can be an important tool for surgeons to innovate surgical methods. However, at this stage, most methods for establishing VVF animal models are surgical^2,3,7^. These methods are complicated, time-consuming, and have low success rates. Therefore, it is imperative to explore a simple, safe, and successful animal disease model of VVF.

Magnetic surgery is a newly developed surgical technique in recent years^8^. Magnetic surgical techniques can be used for digestive tract anastomosis^9–11^, vascular anastomosis^12,13^, and ureterovesical anastomosis^14^. Magnetic anchor technique for laparoscopic cholecystectomy^15^ and magnetic anchor technique-assisted endoscopic submucosal dissection^16,17^ have also seen implementation. Magnetic compression technique (MCT) can also be used for therapeutic fistulas, such as vesicocutaneous (vesicostomy)^18^ and gastrocutaneous fistulas (gastrostomy)^19^.

In terms of animal model preparation, we previously used MCT to establish an animal model of tracheoesophageal fistula in Beagle dogs^20^, which yielded the advantages of non-invasiveness, simplicity, and high success rate. Accordingly, in this study, we proposed the idea of using MCT to establish a VVF animal model and verified the feasibility of this hypothesis using Beagle dogs as model animals.

## Materials and methods

### Ethics statement and animals

The experimental protocol was approved by the Committee for Ethics of Animal Experiments of Xi’an Jiaotong University (Permit number: XJTUAE2023-2208). The research protocol and all the experimental procedures were performed in strict compliance with the Guidelines for the Care and Use of Experimental Animals issued by the Xi’an Jiaotong University Medical Center. And the study is reported in accordance with ARRIVE guidelines. Eight female Beagle dogs (age, 1–3 years; weight, 10–12 kg) were acquired as experimental animals from the Laboratory Animal Center of the Xi’an Jiaotong University (Xi’an, China). No control group was set up in this study, and all experimental animals were included in the study group. The animals were acclimatized to laboratory conditions (23°C, 50% humidity, 12 h:12 h light–dark cycle, and food and water provided ad libitum) for one week before commencing the experiments.

### Magnets

**Figure 1:**
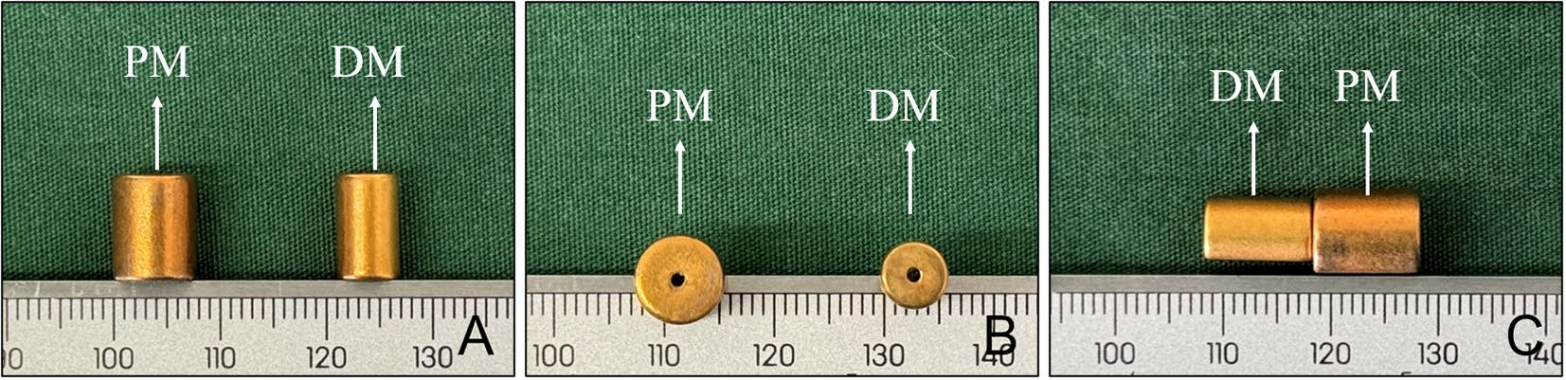
Physical drawing of DM and PM. (**A**) Side view of the PM and DM. (**B**) Bottom view of the magnets. (**C**) Side view when the two magnets attract each other.

In this study, a pair of cylindrical magnets, including a parent magnet (PM, 8 mm diameter) and a daughter magnet (DM, 6 mm diameter), were used to establish the animal model of VVF. Both magnets had a height of 10 mm and had a central hole with a diameter of 1 mm along their *z* axes. The magnets were made of N50 sintered NdFeB permanent magnet material, with saturation magnetization along their *z* axes and titanium nitride coating on the surface (Fig. 1A–C). The mass of the DM and PM was 2.06 g and 3.68 g, respectively, and their magnetic field intensity was 3600 GS and 4200GS, respectively. The magnetic force between the magnets at zero distance was 36.52 N.

### Operation design

**Figure 2:**
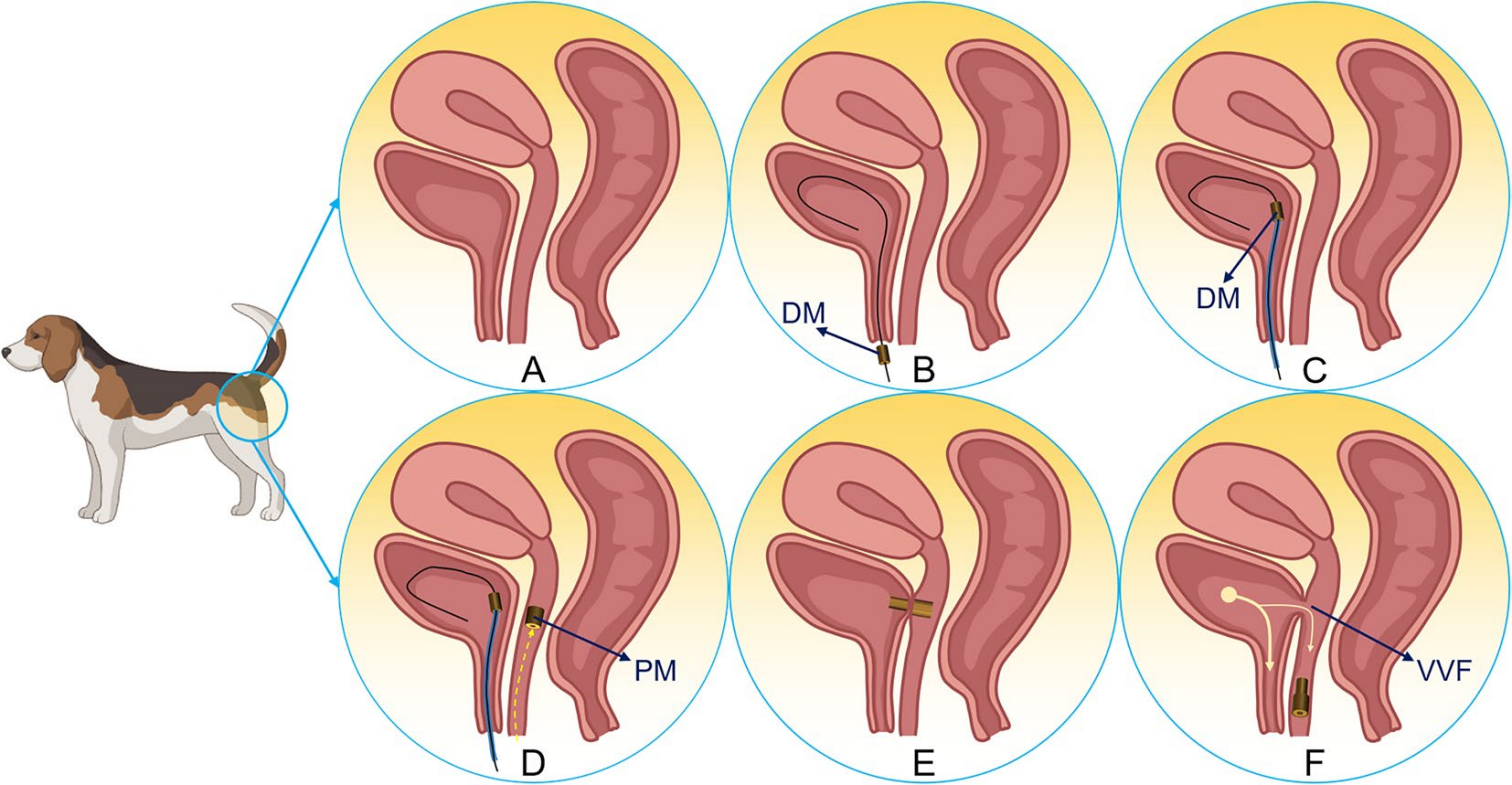
Schematic drawing of the interventional procedure and removed the magnets. (**A**) The normal anatomy of the bladder and vagina. (**B**) A zebra guidewire is inserted into the bladder. (**C**) The plastic catheter pushes the DM along the zebra guidewire into the bladder. The guidewire was inserted through the urethra into the bladder. (**D**) The PM is inserted through the vagina. (**E**) The DM and PM attract each other. (**F**) The attracted magnets were both removed with the help of a vascular clamp through the vagina at once, leaving an open VVF.

The procedure was performed in a non-surgical manner. After anesthesia, the dogs were fixed in a supine position, exposing the urethra and vagina (Fig. 2A). The head of the zebra guidewire was inserted manually into the bladder through the urethra, and the DM was inserted through the tail of the zebra guidewire via the central hole (Fig. 2B). Then, a piece of plastic tube was introduced and advanced through the tail of the zebra guidewire; this tube was used to push the DM into the bladder along the zebra guidewire (Fig. 2C). Then the PM was held into the vagina using a vascular clamp (Fig. 2D), and the position of the DM and PM was observed under X-ray. The position of the two magnets was adjusted such that they attracted each other and compressed the dorsal wall of the bladder and the ventral wall of the vagina as a consequence of this attraction (Fig. 2E). Two weeks after operation, remove the magnets through the vagina, thus establishing a fistula between the bladder and vagina (Fig. 2F).

### Operation procedure

**Figure 3:**
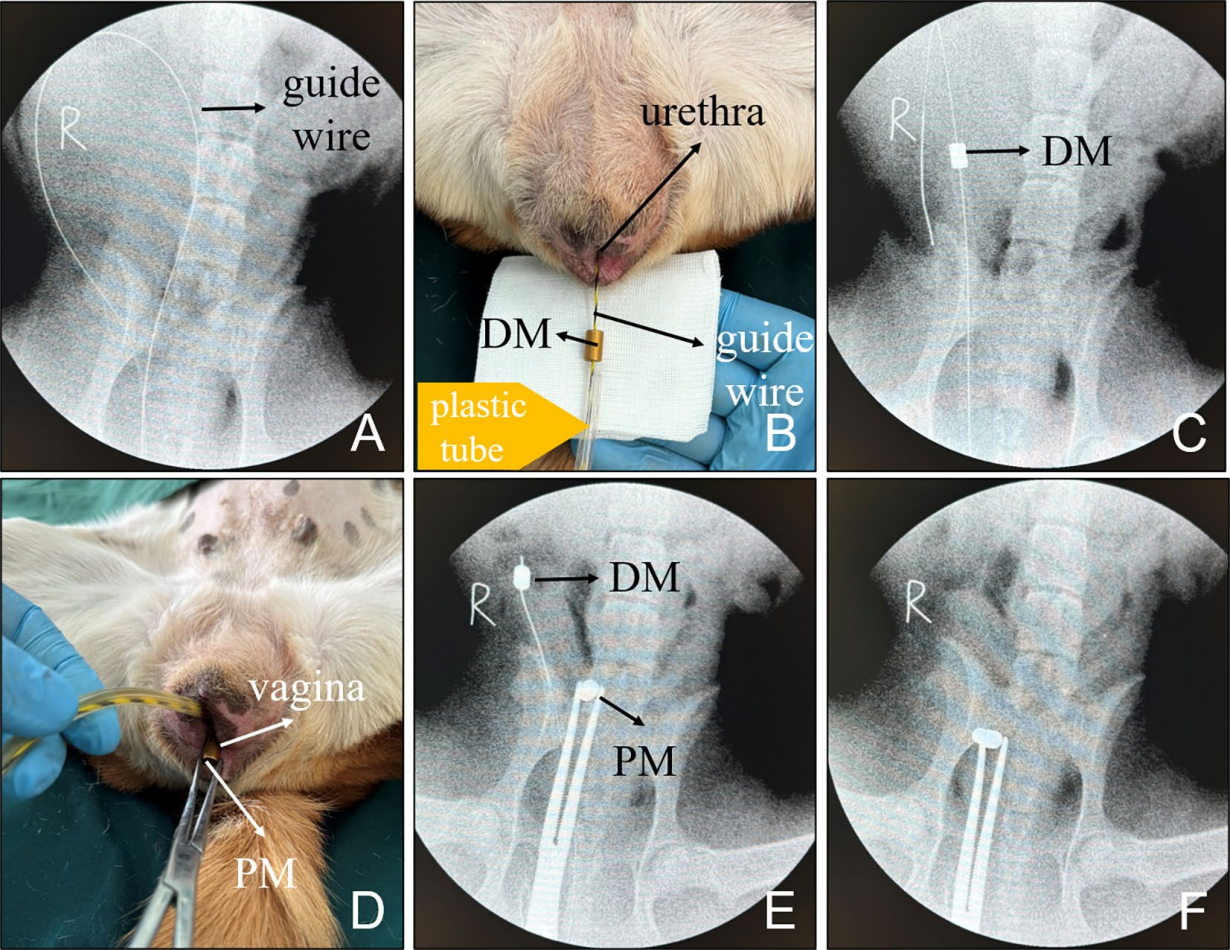
Representative images taken during the operation. (**A**) X-ray after the guidewire entered the bladder. (**B**) The DM followed by the plastic tube is inserted through the tail end of the guide wire. (**C**) The plastic catheter pushes the DM into the bladder. (**D**) The PM is inserted vaginally using a vascular clamp. (**E**) The position of the magnets is observed under X-ray. (**F**) The magnet position is adjusted such that the DM and PM are attracted to each other.

Dogs were fed for one week after purchase from the Laboratory Animal Center of the Xi’an Jiaotong University. They underwent fasting for 6 h and water deprivation for 2 h before intervention. The dogs were anesthetized by intravenous injection of 3% pentobarbital sodium (1 mL/kg) and fixed on the operating table in a supine position. The urethra and vagina were fully exposed, and the zebra guidewire was inserted into the bladder through the urethra. X-ray examination was used to confirm that the zebra guidewire was located in the bladder (Fig. 3A). Then, the DM and a plastic catheter were inserted and advanced through the tail of the zebra guidewire, and the plastic catheter was used to push the DM along the zebra guidewire and advance it into the bladder (Fig. 3B). X-ray examination was used to confirm that the DM had entered the bladder (Fig. 3C). Then, while ensuring that the zebra guidewire and the plastic catheter remained stationary, a vascular clamp was used to insert and advance the PM through the vagina (Fig. 3D). X-ray examination was used to confirm the proper positioning of the PM in the vagina while observing the relative locations of the DM and PM (Fig. 3E). Then, magnet positions were adjusted to allow the DM and PM to attract each other (Fig. 3F). Then, the zebra guidewire and plastic catheter were removed, and the vascular clamp was removed as well, indicating the end of the operation. The operation time was recorded, and any bleeding documented of the urethra and vagina was observed.

### Postoperative care

**Figure 4:**
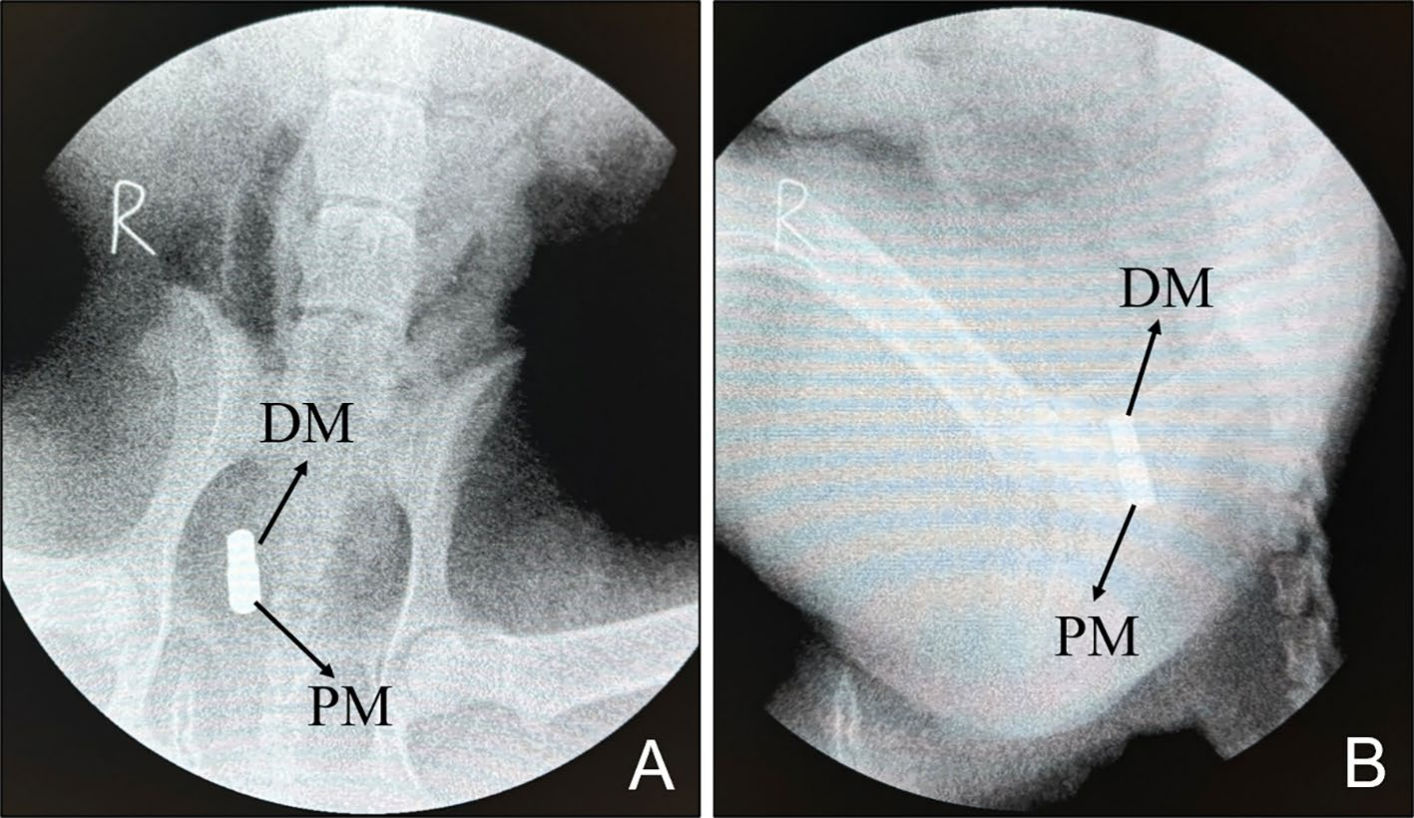
Postoperative X-ray examination. (**A**) Ventrodosal radiograph. (**B**) Lateral radiograph.

All dogs underwent ventrodosal and latero-lateral X-ray examinations immediately after intervention to determine the position of the magnets (Fig. 4). After recovering from anesthesia, the dogs were kept in a single cage and given ad libitum access to water and food. The general condition of the all dogs was observed after the operation every day for 2 weeks. X-ray examinations were performed every other day to observe the position of the magnets.

### Cystography

Two weeks after the operation, the all dogs were anesthetized again with the same protocol as the previous procedure. The position of the magnet was observed by X-ray. The vascular clamp was inserted through the vagina; then, the magnets were attracted to the head end of the vascular clamp and removed along with the clamp when the clamp was pulled out. Cystography was performed by inserting a balloon catheter through the urethra to observe for iohexol (20 ml, 10 ml: 3 g) leakage into the vagina.

### Tissue harvest and analysis

The dogs were euthanized by rapid intravenous injection of high-dose 3% pentobarbital sodium solution (2 mL/kg). The pelvic cavity was carefully dissected to obtain the whole organ cluster of the reproductive system and lower urinary tract. The VVF was carefully dissected to find the dense adhesion between the bladder and vagina. Gross specimens of the VVF were obtained; the fistulas were identified, and its diameter was measured. Then, anastomotic specimen was soaked overnight in 10% formalin for fixation. Thereafter, the specimen was embedded in paraffin, and a 4 μm-thick section from the anastomosis was prepared. The sections were stained with hematoxylin and eosin (H&E) and Masson trichrome and examined under a bright-field microscope.

### Statistical analysis

SPSS statistical 20.0 software was used for data analysis. The quantitative data of normal distribution were described as mean ± SD, and non-normally distributed data were presented as the median.

## Results

### Procedural parameters

The two magnets were successfully placed in all the Beagle dogs; the magnets automatically attracted and aligned well, and no bloody discharge was observed in the urethra or vagina in any of the dogs. The average operation time was 14.38 min ± 1.66 min (range, 12–17 min).

### Survival rate and postoperative adverse events

Two weeks postoperatively, the survival rate of all dogs was 100%. The dogs were generally in good condition after the operation, with no bleeding, dysuria, or other adverse events.

### Magnet removal and cystogram

**Figure 5:**
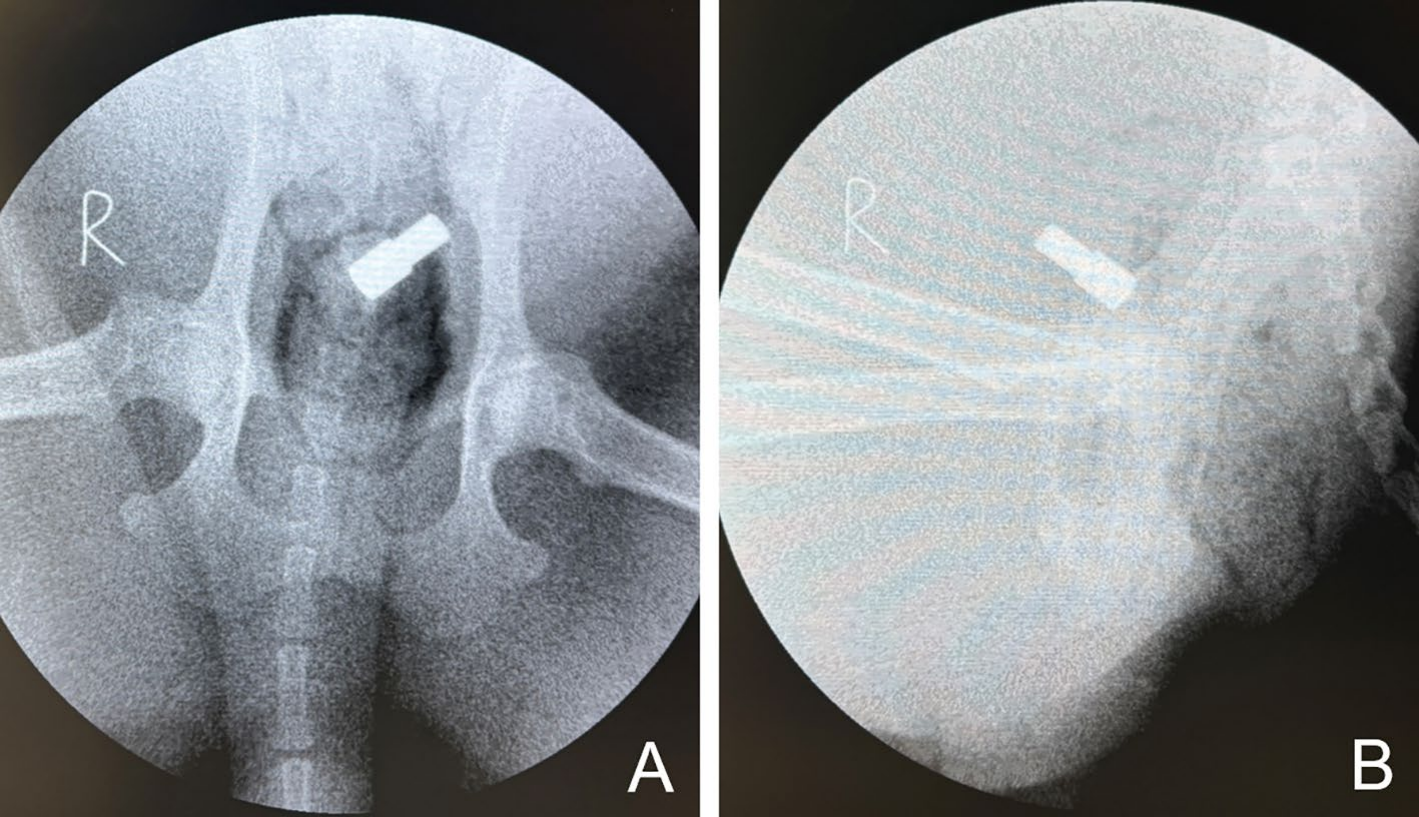
X-ray examination performed 2 weeks postoperatively showed the location of the magnet. (**A**) Ventrodorsal radiograph. (**B**) Lateral radiograph.

**Figure 6:**
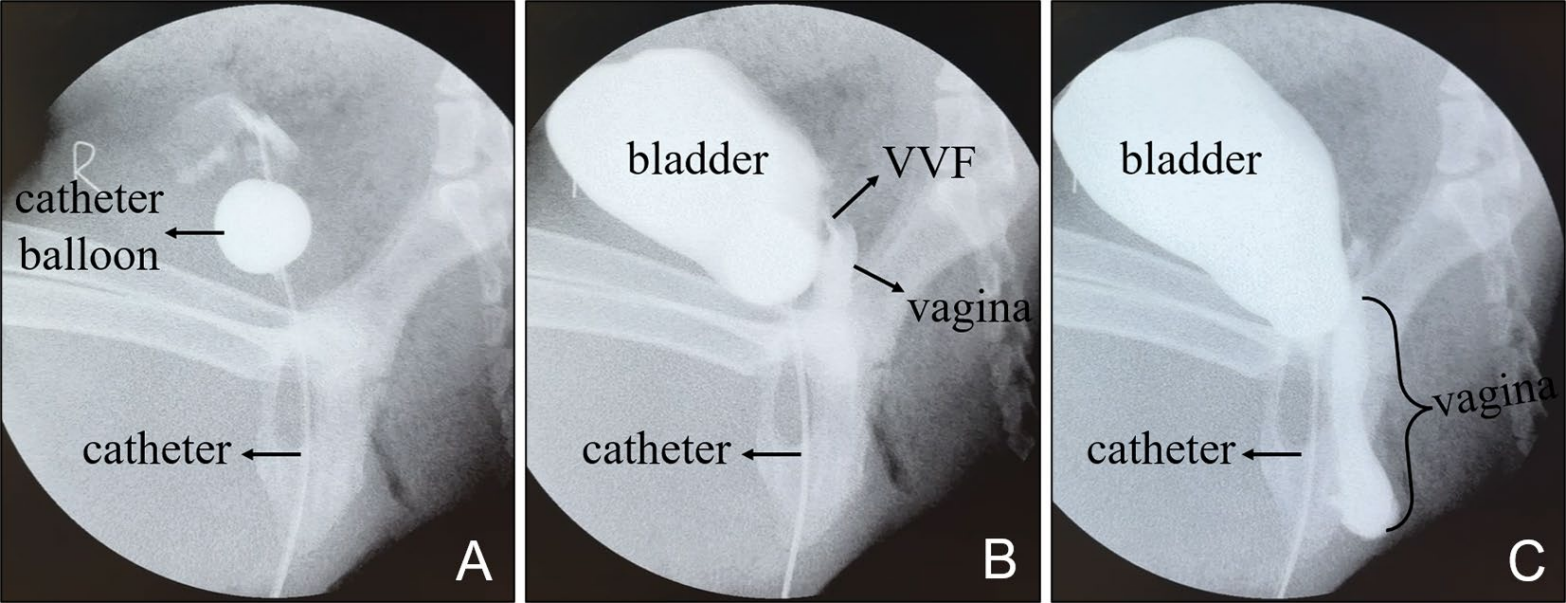
Cystography. (**A**) A balloon catheter was inserted into the bladder. (**B**) Vesicovaginal fistula and the upper vaginal segment were observed when the contrast medium was injected. (**C**) Contrast media can be seen in the vagina.

X-ray examination performed 2 weeks postoperatively indicated that the magnets were retained in the body (Fig. 5). All the magnets in the dogs were easily pulled out through the vagina. The dogs were placed in the right lateral position with the hind legs extended position and injected with an appropriate amount of contrast agent (iohexol solution) through the urinary catheter (14Fr). Then, the leakage of the contrast agent into the vagina was visualized by cystography, and a VVF could be seen between the ventral wall of the vagina and the dorsal wall of the bladder (Fig. 6).

### Gross and histological appearance of anastomosis

**Figure 7:**
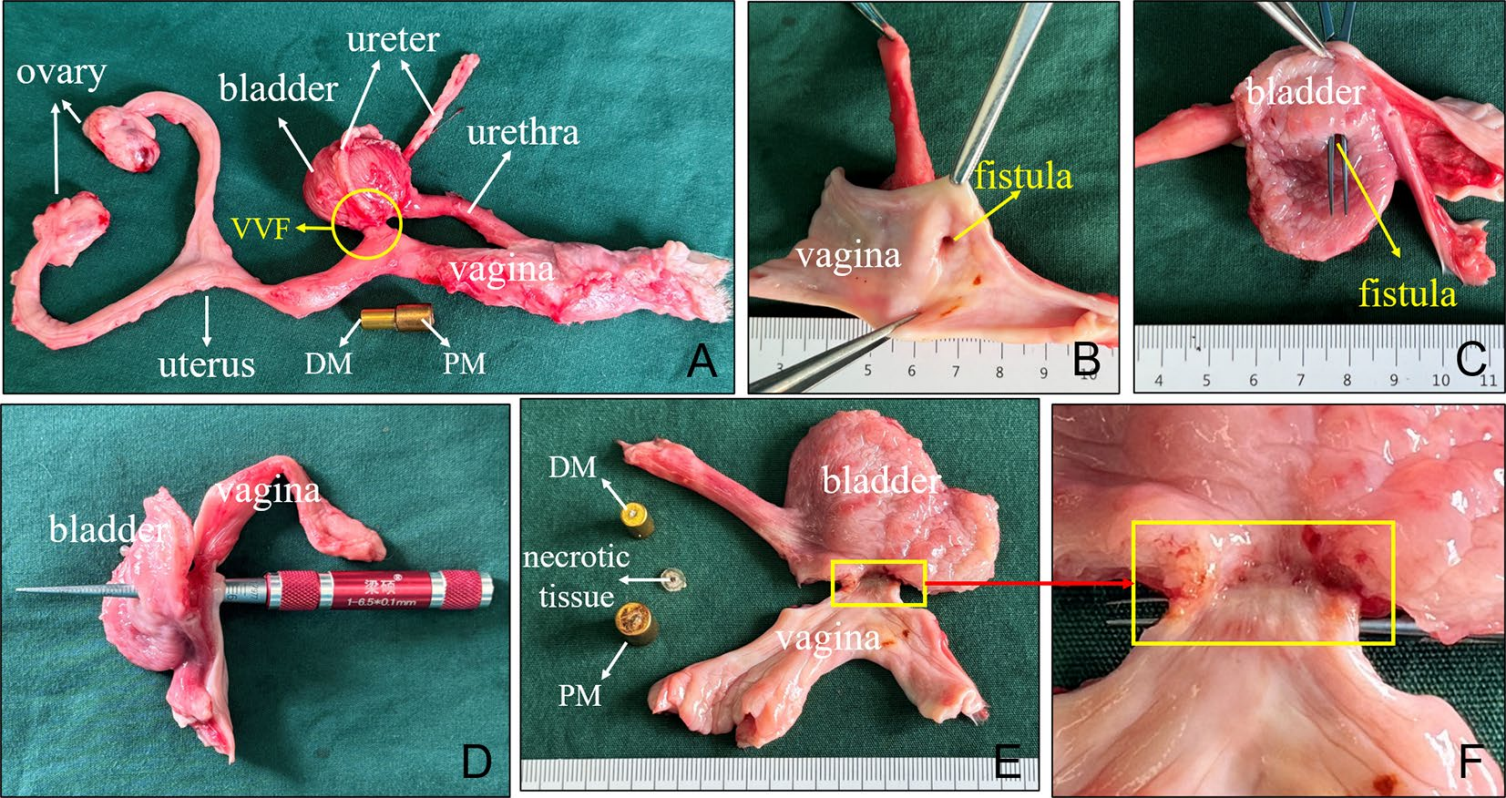
Gross specimens of the vesicovaginal fistula. (**A**) Whole-specimen view of the vesicovaginal fistula. (**B**) Vaginal opening of VVF. (**C**) Vesical opening of VVF. (**D**) The diameter of the fistula was measured. (**E**, **F**) A lateral view of the inside of the fistula, the removed magnets, and the necrotic tissue between the magnets.

**Figure 8:**
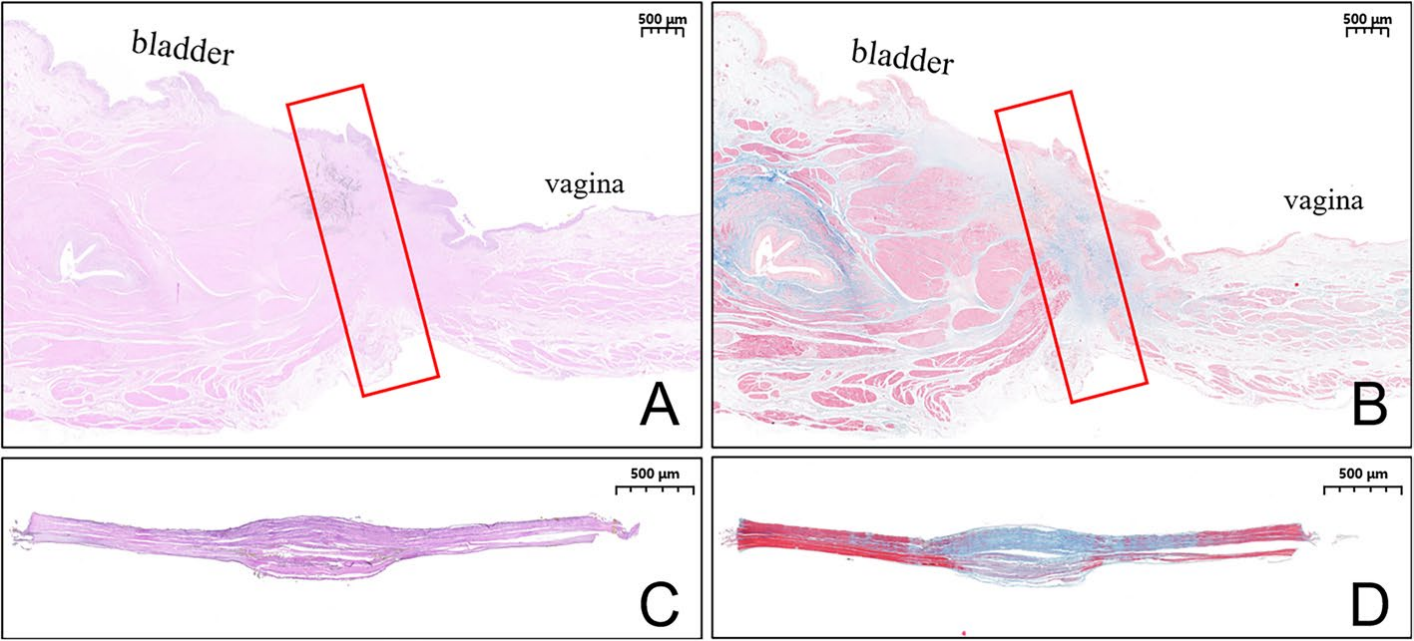
Histological specimens. (**A**, **B**) Histological observation of the vesicovaginal fistula (1.6 ×). (**C**, **D**) Necrotic tissue between the magnets (3.0 ×).

The gross specimens showed that the fistula formed between the posterior wall of the bladder and the anterior wall of the vagina, and the surrounding tissue of the fistulas showed no adhesion, inflammation, or edema (Fig. 7A). When the vagina was dissected longitudinally, the vaginal fistula was clearly visible (Fig. 7B), and the vaginal mucosa around the fistula was smooth, indicating successful formation of the fistula. The fistula could be seen in the posterior wall of the bladder (Fig. 7C). The diameter of the fistula was measured by the conical bore gauge (measuring range is 1.0–6.5 mm) is 5.41 mm ± 0.72 mm (range, 4.50–6.24 mm; Fig. 7D). The VVF was dissected longitudinally. The lateral wall of the vagina and the lateral wall of the bladder appeared closely connected, and the inner surface of the fistula was smooth (Fig. 7E,F). Necrotic tissue (bladder wall and vaginal wall) between magnets was visible after separating the magnets from each other (Fig. 7E). H&E and Masson staining showed the continuity between the vaginal mucosa and the bladder mucosa has been established at the fistula (Fig. 8).

## Discussion

In this study, a canine VVF model was successfully established using MCT. Both gross specimens and histology showed that the fistula was well formed. At present, MCT is mainly used for digestive tract anastomosis. In gastrointestinal anastomosis, the compressed tissue between the magnets underwent ischemia, necrosis, and shedding, whereas the tissue adjacent to the compressed tissue between the magnets completed the histopathological process of adherence, repair, and healing^8,21^. Zhang et al.^21^ established the staging (Yan-Zhang’s staging) of digestive tract magnetic anastomosis using a rat colonic anastomosis model. The combination of MCT and endoscopic technique has realized the nonsurgical reconstruction of the digestive tract^22,23^, which is particularly beneficial in the treatment of digestive tract stenosis^24^.

The ability of adjacent magnets to generate force without direct contact makes magnetic materials valuable in medicine. When two magnets are located in cavities of two adjacent organs, the two cavity organs are pulled because of magnetic attraction, and a magnet-tissue-magnet sandwich structure is formed at the site of magnetic attraction; the compressed tissue undergoes ischemia, followed by shedding of the necrotic tissue, and a new channel is finally established here. This is the theoretical basis for the establishment of VVF by MCT.

This study cleverly introduced magnetism into the establishment of animal models of disease. This study has the following strengths: (1) The surgical operation is replaced by a non-invasive operation, which greatly reduces the difficulty of model preparation and reduces the trauma to animals. (2) The diameter of the fistula is almost the same as the outer diameter of the DM, and thus, fistulas of different sizes may possibly be established by changing the diameter of the DM. It is easier to unify the location and size of the fistula in animal models of VVF established by MCT. (3) The establishment of a VVF by MCT is equivalent to the side-to-side anastomosis between the bladder and the vagina, and the continuity of the bladder mucosa and vaginal mucosa on the inner surface of the fistula formed by this method maybe good, which can avoid the problem of fistula self-closure in experimental animals. (4) The individual height of both magnets used in this study was 10 mm, resulting in a total magnet length of 20 mm after alignment; given the narrow lumens, this total length may explain why the magnets stayed in place and could not exit the body even after the establishment of VVF. However, this should not discourage further research on VVF. In this experiment, if spontaneous voiding of magnet is desired, we could easily remove the magnet using a vascular clamp. If researchers need the magnet to move out of the cavity spontaneously and exit the body by itself, the overall height of the magnet can be reduced to make it easier to exit. In order to ensure that the magnetic force is not affected after lowering the height of the magnet, permanent magnet materials with better magnetic properties can be selected to manufacture the magnet.

Although this study successfully established an animal model of VVF using MCT, it has certain limitations and deficiencies. First, as there was no control group, the superiority of the use of MCT to conventional modeling methods remains unestablished because of no comparative statistical analysis on quantitative indicators between the two methods. Second, the number of experimental animals was small. Third, we did not observe the natural progression of the VVF over a long term.

In summary, MCT is an ideal method to establish a VVF model in Beagle dogs and offers the benefits of simplicity, feasibility, small trauma, and easy standardized control. This model should be further evaluated in a surgical setting to validate it as an option for studying new techniques or materials to boost surgical innovation.

## Data Availability

The data underlying this article will be shared on reasonable request to the corresponding author.
